# Enhanced Antitumoral Activity of Extracts Derived from Cultured *Udotea flabellum* (Chlorophyta)

**DOI:** 10.1155/2011/969275

**Published:** 2011-07-21

**Authors:** Rosa Moo-Puc, Daniel Robledo, Yolanda Freile-Pelegrin

**Affiliations:** ^1^Department of Marine Resources, Cinvestav, Km 6 Carretera Antigua a Progreso, Cordemex, A.P. 73, 97310 Mérida, YUC, Mexico; ^2^Unidad de Investigación Médica Yucatán, Unidad Médica de Alta Especialidad, Centro Médico Ignacio García Téllez, Instituto Mexicano del Seguro Social; 41 No 439 x 32 y 34, Colonia Industrial CP, 97150 Mérida, YUC, Mexico

## Abstract

Very few studies have been performed to evaluate the effect of culture conditions on the production or activity of active metabolites in algae. Previous studies suggest that the synthesis of bioactive compounds is strongly influenced by irradiance level. To investigate whether the antiproliferative activity of *Udotea flabellum* extracts is modified after cultivation, this green alga was cultured under four photon flux densities (PFD) for 30 days. After 10, 20, and 30 days, algae were extracted with dichloromethane: methanol and screened for antiproliferative activity against four human cancer cell lines (laryngeal—Hep-2, cervix—HeLa, cervix squamous—SiHa and nasopharynx—KB) by SRB assay. Lipid and phenol content were evaluated by standardized methods on algae organic extracts. After 10 days of cultivation, organic *U. flabellum* extracts showed a significant increase in antiproliferative activity on Hela and SiHa cells when compared to noncultured algae extracts. Extracts obtained after 10 and 20 days of culture were active on KB and Hep-2 cells. Total phenol and polyunsaturated fatty acid content in organic extracts changed with cultivation time but not by irradiance treatment. Extracts from *U. flabellum* obtained after 10 and 20 days of culture have been selected for fractionation and isolation of active compounds.

## 1. Introduction

Natural products and related drugs are used to treat 87% of all categorized human diseases including bacterial infection, cancer, and immunological disorders [1]. Approximately 25% of prescribed drugs in the world originate from plants [2] and over 3000 species of plants have been reported to have anticancer properties [3]. Recent trends in drug research on natural sources suggest that algae are a promising source of novel biochemical active substances [4]. To survive in a competitive environment, marine algae have developed defense strategies that result in a significant level of structural chemical diversity that is derived from different metabolic pathways [5]. The effect of culture conditions on the production or activity of active metabolites in algae has scarcely been studied and consequently remains poorly understood. In other alga models, such as the cyanophyte *Scytonema*, increasing irradiance gradually increased antibiotic production [6]. Similarly, *Spyridia filamentosa*, a red alga cultured at different light irradiances, had contrasting antibiotic activities that were strongly influenced by irradiance level [7]. Most recently, extracts obtained from *Penicillus dumetosus *cultured at different light irradiances displayed varying antiproliferative activity against diverse cancer cell lines [8]. The feasibility of algal cultivation can help to induce adaptations that can be measured through metabolite synthesis or biological activity. Fully controlled greenhouse-based cultivation systems have been developed for high-quality year-round vegetable production for the botanical drug market [9]. Therefore, a better understanding of the potential manipulation of algal culture conditions to modify metabolite synthesis and activity is required. 

 Tropical green algae in the order Bryopsidales, including those of the genera *Avranvillaea*, *Caulerpa*, *Halimeda*, *Penicillus*, and *Udotea*, are noted for the production of sesqui and diterpenoids, compounds that have also shown antifungal and antiproliferative activity [5, 10]. Recent studies have shown that both aqueous and organic extracts of *Udotea flabellum* exhibit in vitro antiprotozoal [11, 12] as well as cytotoxic and antiproliferative activities on cancer cell lines [13]. In some cases, the antiproliferative activity of marine algae extracts has been positively correlated with total polyphenol content, suggesting a causal link between the extract content of polyphenols and phenolic acids [14], while other authors have reported a variety of fatty acids and derivatives with antiproliferative effects in different cancer cell models [15]. Despite the observations of antiproliferative activity in marine algae, there is limited information on how this activity may change under contrasting environmental conditions. Therefore, the objective of this study was to investigate the antiproliferative activity of crude organic extracts of cultured *Udotea flabellum* on four human malignant cell lines (HeLa, Hep-2, SiHa, and KB) and their change over time under four light treatments. Furthermore, the study evaluated whether phenol content and lipid composition were related to its antiproliferative activity.

## 2. Materials and Methods

### 2.1. Alga Collection and Culture Conditions

**Figure 1:**
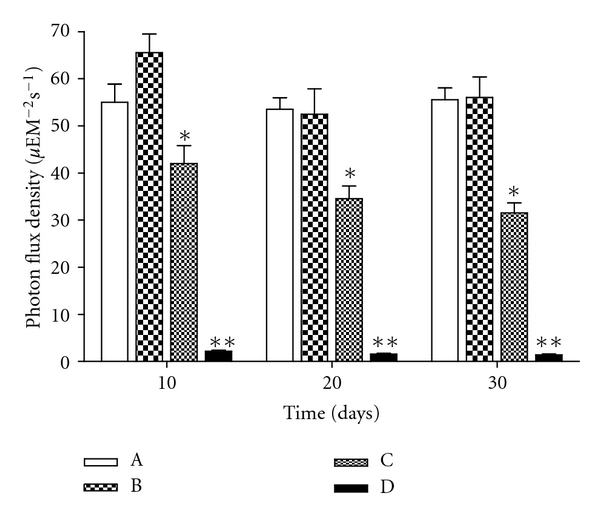
Photon flux density during cultivation of *U. flabellum*. (A) full sunlight (100 %), (B) 75 % sunlight, (C) 50 % sunlight, and (D) 0 % sunlight. Asterisk indicates significant differences.

*Udotea flabellum *(J. Ellis and Solander) M. A. Howe were collected along the YUC Peninsula coast, Mexico, stored in plastic bags and chilled in ice during transport to the Cinvestav Marine Station at Telchac, Yucatan, Mexico. Algae were cultivated under four light treatments: full (100%) sunlight, 75% sunlight, 50% sunlight, and 0% sunlight, designated treatment A, B, C, and D, respectively. Agricultural greenhouse shade net was used in order to obtain variable light intensities in the culture system. Light intensity varied over cultivation time: during the first 10 days, the photon flux density (PFD) in full sunlight and 75% sunlight treatments were not significantly different (one-way ANOVA, *F*[3,36] = 68.21, *P* < 0.0001; post hoc Tukey's test *P* < 0.0001) at 55 ± 12.9 and 65 ± 12.5 *μ*mol photon m^−2^s^−1^, respectively, while 50, and 0% treatments received 60, and 3% of incident PFD, respectively (42 ± 12.0 and 2 ± 0.7 *μ*mol photon m^−2^s^−1^). After 20 and 30 days of cultivation, a similar trend was registered, with the 75% treatment receiving 75–100% of incident PFD and the 50% treatment receiving 42–52%; the 0% treatment only received 2% of incident PFD (Figure 1).

### 2.2. Preparation of Extracts

Freshly collected samples of the wild material were lyophilized and ground into powder to perform plant extraction protocols and analytical methods; this material was considered as a control before cultivation (time 0). Entire plants (*n* = 15) were taken from each culture treatment—A, B, C, and D—at 10, 20, and 30 days into the experimental period to perform organic extraction and analysis. Lyophilized samples (20 g) were exhaustively extracted with 200 mL of dichloromethane: methanol (7 : 3) by maceration for 24 h at room temperature. These extracts were filtered and concentrated to dryness in vacuum at 40°C and stored at −20°C until required. Every extract was labeled according to culture conditions: light intensity (A, B, C, or D) and time (10, 20, and 30 days).

### 2.3. Chemicals

Dulbecco's Modified Eagle's Medium (DMEM), heat-inactivated fetal bovine serum (FBS) and penicillin and streptomycin (PS) were purchased from Gibco, USA. 3-(4,5-dimethylthiazol-2-yl)-2,5-diphenyl tetrazolium bromide (MTT), Dimethyl sulfoxide (DMSO), sulforhodamine B (SRB) and trichloroacetic acid (TCA) were obtained from Sigma.

### 2.4. Cell Culture

The following cell lines were used for the antiproliferative assays: normal Mardin-Darbin cell kidney (MDCK), and four human carcinoma cells, namely, laryngeal (Hep-2), cervix (HeLa), cervix squamous (SiHa) and nasopharynx (KB). The cells were grown in DMEM media supplemented with 10% v/v fetal bovine serum (FBS) with 100 U mg mL^−1^ of PS. Cell lines were maintained at 37°C in a 5% CO_2_ atmosphere with 95% humidity, and the culture medium was changed once every 5 days.

### 2.5. Cytotoxicity Assay

The cytotoxicity assay was performed according to Rahman et al. [16], where 1.5 × 10^4^ viable cells from each cell line were seeded in a 96-well plate and incubated for 24 to 48 h. When cells reached >80 % confluence, the medium was replaced and the cells were treated with the organic extracts dissolved in dimethyl sulfoxide (DMSO at a maximum concentration of 0.05%) at 6.25, 12.5, 25, and 50 *μ*g mL^−1^. After 72 h of incubation, 10 *μ*L of MTT solution (5 mg mL^−1^) was added to each well and incubated at 37°C for 4 h. The medium was removed and formazan, generated by the activity of dehydrogenases, was dissolved in acidified isopropanol (0.4 N HCl). The amount of MTT-formazan generated is directly proportional to the number of living cells and was determined by measuring the optical density (OD) at 540 nm using a Bio-assay reader (BioRad, USA). Untreated cells were used as a negative control. The concentration of the organic extract that killed 50% of the cells (CC_50_) was calculated with GraphPad-PRISM 4.00 software. All determinations were performed in triplicate.

### 2.6. Antiproliferative Assay

For the antiproliferative assay, we used sulforhodamine B (SRB), a colorimetric assay which estimates cell number by staining total cellular protein with the SRB dye, in order to assess cell growth inhibition [16]. This method used the same conditions as the cytotoxic assay except that the medium was replaced with DMEM 10% FBS to induce cellular proliferation during extract treatments. After 48 h incubation, the medium was discarded and cells were fixed with 100 *μ*L of ice-cold 40% TCA. Thereafter, the cells were incubated at 4°C for 1 h and the plates were washed five times with cold water. The excess water was drained off and the plates were left to dry; 50 *μ*L of SRB stain (10 mg w/v in 1% acetic acid) was added to each well for 30 min. Finally, the plates were washed with 50 mL of 1% acetic acid and rinsed four times until dye adhering to the cells was observed. The OD was measured at 540 nm using a microplate reader (model 450, Bio-Rad, USA). Untreated cells were used as a negative control. Docetaxel, a clinically well-established antimitotic chemotherapy medication was used as a positive control of antiproliferative activity. The IC_50_ value, that is, the concentration of organic extract that produced a 50% reduction in the surviving fraction, was calculated using GraphPad-PRISM 4.00 software. MDCK cell line was used to evaluate the selective index (SI) of *U. flabellum *extracts. SI is defined as the ratio of cytotoxic to antiproliferative activity. All determinations were performed in triplicate.

### 2.7. Phenolic Content

Total phenolic content of the algal extracts was determined spectrophotometrically using Folin-Ciocalteu reagent [17]. First, 20 mg of the dry extract was diluted with methanol (3 mL). Aliquots of the diluted extracts (0.1 mL) were transferred into the test tubes; 2.9 mL of distilled water and 0.5 mL of Folin-Ciocalteu reagent were added. After 10 min, 1.5 mL of 20% sodium carbonate solution was added, mixed thoroughly and allowed to stand at room temperature in the dark for 1 h. Absorbance was measured at 725 nm and total phenolic content (expressed as % of dry weight) was calculated based on a standard curve of phloroglucinol.

### 2.8. Lipid Content

Total lipids were determined according to a previously reported method [18]. The algae extract (20 mg) was homogenized with a mixture of H_2_O, methanol and chloroform (1 : 1 : 9 v/v). The chloroform layer containing dissolved lipids was collected, dried with nitrogen, and saponified with 1.2 M NaOH. Fatty acids were converted to methyl esters with 0.6 mL of 10 M HCl and 1 mL of 12% boron trichloride in methanol at 80°C for 60 min. After methylation, 1 mL of hexane: diethylether (1 : 1) and 3 mL of 0.3 M NaOH were added, and the resultant mixture was dried with nitrogen and recovered with hexane. The total content of fatty acid methyl esters was analyzed by gas chromatography (Hewlett Packard 6890 Plus with Supelco SP2560 bis-cyanopropyl polysiloxane capillary column 100 m × 0.25 mm × 0.25 *μ*m internal diameter). The column temperature programming was set from 140 (5 min) to 240°C (20 min) at a rate of 4°C  min^−1^. Injector and detector temperature was 260°C. Helium was used as the carrier gas at a flow rate of 1.1 mL min^−1^. Fatty acid methyl esters were identified by comparing their retention times with those of standard samples. The lipid analyses were carried out in duplicate, and the results expressed as percentages of algae extract dry weight (% dry wt).

### 2.9. Statistical Analysis

Data were analyzed with GraphPad 4.0 Software Inc. (San Diego, Calif, USA). The dose-response curves (variable slope) were fitted with the algorithm: *Y* = *E*_min_ + [(*E*_max __*E*_min_)/(1 + 10(Log ED50 − Log D)  Hill  slope)]. Statistical analysis was performed with parametric tests because variances were homogeneous between groups. An unpaired Student's *t*-test (two-tailed) was applied when only two groups were compared. A one-way ANOVA followed by post hoc Dunnett's test was used to assess the differences when three or more groups were simultaneously compared. Values in text and figures are expressed as means ± SD.

## 3. Results

### 3.1. Antiproliferative Activity

The antiproliferative activity of *U. flabellum *extracts on the growth of four cancer cells *in vitro* are summarized in Table 1. The organic extract of wild *U. flabellum *collected (time 0) showed antiproliferative activity (IC_50_) on SiHa (276.2 ± 1.9 *μ*g mL^−1^), HeLa (296.6 ± 0.9 *μ*g mL^−1^), Hep-2 (52.9 ± 1.0 *μ*g mL^−1^), and KB (47.8 ± 1.2 *μ*g mL^−1^). After10 and 20 days of cultivation, the antiproliferative activity of the *U. flabellum *extracts on the SiHa cell line significantly improved when compared with non cultured *U. flabellum* extracts (IC_50_ 276.2 *μ*g mL^−1^), with a reduction of 85–95% after 10 days and 63–77% after 20 days of cultivation. The antiproliferative activity of extracts on the HeLa cell line had the same tendency, with a 70–90% reduction of the non cultured *U. flabellum* extracts IC_50_; whereas extracts obtained after 30 days of culture increased IC_50_ by approximately 78–185% and 40–95% on the SiHa and HeLa cell lines, respectively. For the Hep-2 and KB cell lines *U. flabellum* extracts only showed a 36–69% and 40–51% reduction of IC_50_ after 10 days of cultivation. 

In general, *U. flabellum* extracts obtained after cultivation showed improved SI on cancer cells, particularly the extracts from culture treatment A (10 days), which showed the highest selectivity index ranging from 6–28 and 8–20 on the SiHa and Hep-2 cell lines, respectively (Table 2).

### 3.2. Phenol and Lipid Content

**Figure 2:**
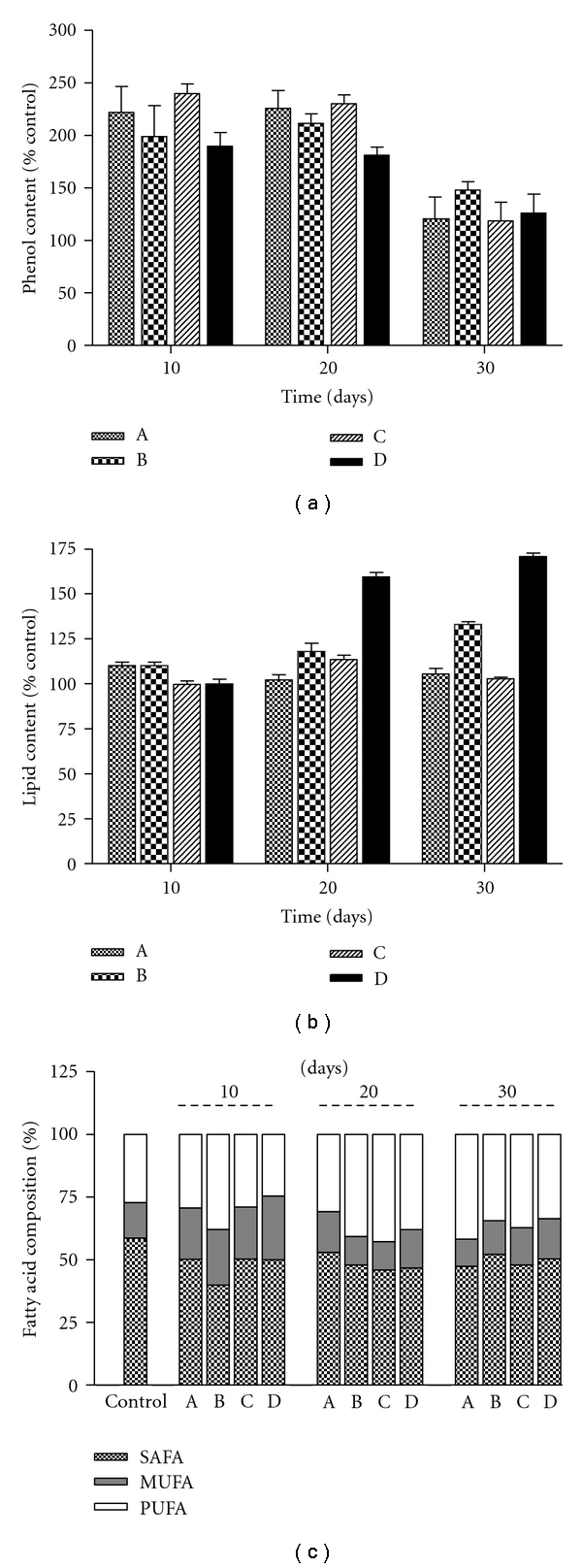
Phenol and lipid content of* Udotea flabellum *extracts. (a) Total phenol content expressed as a percentage of the control (%), (b) lipid content in organic extracts expressed as a percentage of the control (%), (c) fatty acid composition in organic extracts expressed in % dry weight (d wt) over the culture period. Each symbol is the mean±SD of three assays, normalized with the control extract collected at the same time and place without culture treatment. Time when the samples were collected from the culture tank: 10, 20, and 30 days.

*U. flabellum* extracts before cultivation (time 0) showed a total phenol content of 1.7 ± 0.2% dry wt. *U. flabellum* extracts increased phenolic content by 100% for all treatments after 10 and 20 days of cultivation (Figure 2(a)). Lipid and fatty acid content in *U. flabellum *extracts varied in relation to time and light availability (Figure 2(b)). *U. flabellum *extracts before cultivation (time 0) showed a total lipid content of 46.4 ± 1.5%, increasing by 60 and 70% after 20 and 30 days of cultivation without light. GC analysis revealed that saturated fatty acids (SAFA) were dominant (58.6%) while monounsaturated fatty acid (MUFA) and polyunsaturated fatty acid (PUFA) were present at 14.2% and 27.1%, respectively (Figure 2(c)). In general, after cultivation SAFA content decreased by 6–19% in relation to the control while MUFA content remained similar throughout time and cultivation treatment. On the other hand, PUFA content increased 3–15% in plants subjected to culture treatments when compared to the control. The predominant SAFA was palmitic acid (16 : 0), while in the MUFA fraction palmitoleic acid (C16 : 1) and oleic acid (C18 : 1) predominated.

## 4. Discussion

**Figure 3:**
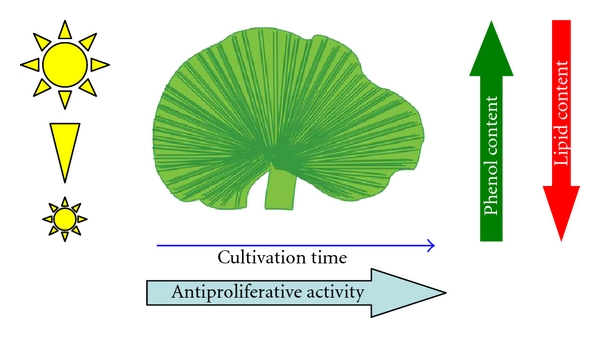
Effects of culture conditions and light on *Udotea flabellum* chemical composition and their role in the antiproliferative activity in cancerous cell lines *in vitro*. Increasing light promotes an increase in phenolic compounds, whereas, lipid content decreases. Therefore, algae extracts increases antiproliferative activity for SiHa, HeLa, Hep-2 and KB cells with time under controlled culture conditions.

Under experimental culture conditions the biological activity of the* U. flabellum *organic extracts improved, showing an increased inhibitory effect with a reduction in the initial IC_50_, thus improving the effect of culture conditions on biological activity (Figure 3). *U. flabellum* extracts obtained after cultivation also improved SI on cancer cells, particularly on the SiHa and Hep-2 cell lines. According to the American National Cancer Institute, the IC_50_ limit in order to consider a crude extract promising for further purification is <30 *μ*g mL^−1^ [19]. Considering the above-mentioned criterion, after 10 days of cultivation all *U. flabellum *extracts increased their activity against SiHa, Hep-2 and KB cells. 

*U. flabellum* extracts increased phenolic content by 100% for all treatments after 10 and 20 days of cultivation. In this study, the inhibition of cancer cell proliferation by *U. flabellum *extracts could not be explained solely by the concentration of polyphenolic compounds. This suggests that other phytochemicals may play a major role in the antiproliferative activity of *U. flabellum *extracts. Previous studies on tropical green algae of the family Udoteaceae have isolated sesquiterpenoids and diterpenoids with biological activity [20] and udoteatrial hydrate with moderate antimicrobial activity against *Staphylococcus aureus* [21]. 

Phenolic compounds have been under study as potential therapeutic agents against a wide range of diseases including neurodegenerative diseases, cancer, diabetes, cardiovascular dysfunction, inflammatory diseases, and aging [22]. In some cases the antiproliferative activity of alga extracts has been positively correlated with total polyphenol content, suggesting a causal link between extract content of polyphenols and phenolic acids [14]. This causal link has also been found with the cytotoxicity *in vitro* of some red and brown algae extracts [23, 24]. Moreover, phenolic compounds including phlorotannins can induce oxidative stress in cancer cells [25]. The above could explain the ability of some algae extracts, such as those from *Lithothamnion calcareum*, to suppress colon polyp formation in mice [26].

In general, *U. flabellum* extracts had high levels of PUFA (linoleic acid and alpha linoleic acid). A variety of fatty acids and derivatives have antiproliferative effects in different cancer cell models [15]. Despite the amount of useful information derived from lipid class analyses in algal physiology, most of the studies available are restricted to microalgae, and little is known about lipid class changes in macroalgae during cultivation.

## 5. Conclusions

The results of this study indicate that the metabolism of a number of active compounds of *U. flabellum *are substantially influenced by culture time but not light treatments, and hence have an influence on the production of metabolites that render them biologically active. Extracts obtained from *U. flabellum *after 10 and 20 days of culture under conditions described in this study showed increased antiproliferative activity on SiHa, HeLa, Hep-2 and KB cells. Although we observed that culture time influenced phenol and lipid content, the compound responsible for the antiproliferative activity of *U. flabellum* extracts remains to be identified.

## Figures and Tables

**Table 1:** Growth inhibition (IC_50_) of *U. flabellum *extracts on cancer cell lines (*P* ≤ 0.001).

Cancer cells	IC_50±_SD (*μ*g mL^−1^)			
	Time = 0	Day = 10	Day = 20	Day = 30
**SiHa**	276.2 ± 1.91			
A		30.4 ± 3.9	100.6 ± 2.65	493.1 ± 2.1
B		42.7 ± 0.9	75.8 ± 1.1	623.3 ± 2.7
C		10.2 ± 1.2	64.5 ± 1.5	788.9 ± 1.2
D		13.1 ± 2.7	62.4 ± 1.2	547.8 ± 2.1
		*F*(4,10) = 6814	*F*(4,10) = 8113	*F*(4,10) = 24910
Docetaxel	0.32 ± 0.01			

**HeLa**	296.6 ± 0.9			
A		49.7 ± 2.1	45.3 ± 1.3	413.6 ± 0.9
B		39.2 ± 3.1	34.8 ± 1.9	580.4 ± 1.9
C		53.8 ± 2.7	67.4 ± 2.8	568.2 ± 1.2
D		63.8 ± 1.8	76.6 ± 1.8	482.6 ± 0.9
		*F*(4,10) = 7143	*F*(4,10) = 10350	*F*(4,10) = 27690
Docetaxel	0.20 ± 0.04			

**Hep-2**	52.9 ± 1			
A		16.6 ± 1.3	71.7 ± 2.1	98.4 ± 1.2
B		19.1 ± 1.8	60.3 ± 1.8	100.7 ± 1.8
C		16.7 ± 0.9	75.2 ± 1.9	91.5 ± 2.3
D		33.5 ± 1.4	91.1 ± 1.9	106.1 ± 1.6
		*F*(4,10) = 424.1	*F*(4,10) = 203.4	*F*(4,10) = 521.7
Docetaxel	0.08 ± 0.03			

**KB**	47.8 ± 1.2			
A		23.4 ± 1.9	134.6 ± 1.9	106.4 ± 1.5
B		29.8 ± 2.1	138.1 ± 1.5	109.7 ± 1.5
C		32.4 ± 1.7	117.3 ± 1.2	123.4 ± 0 .9
D		28.9 ± 1.2	112.7 ± 1.4	126.7 ± 2.3
		*F*(4,10) = 113.9	*F*(4,10) = 1854	*F*(4,10) = 1269
Docetaxel	0.23 ± 0.07			

IC_50_: half maximal (50%) inhibitory concentration (IC) of organic extracts.

**Table 2:** Cytotoxicity (CC_50_) of *U. flabellum *extracts on normal cell line and selective index (SI) for each cancer cell line.

Time (Irradiance treatment)	CC_50_ MDCK Cells	SI			
		SiHa	HeLa	Hep-2	KB
Time = 0	297.9±11.2				
Day = 10					
A	322.3 ± 8.9	10	6.5	19.4	13.7
B	272.6 ± 4.1	6.4	7.0	14.5	9.3
C	286.6 ± 5.1	28	5.3	17.1	8.8
D	276.9 ± 5.8	21	4.3	8.2	9.6
Day = 20					
A	358.2 ± 2.9	3.6	7.9	5.0	2.6
B	323.3 ± 4.6	4.3	9.2	5.4	2.3
C	375.9 ± 6.7	5.8	5.5	5.0	3.2
D	386.1 ± 7.1	6.0	5.0	4.2	3.4
Day = 30					
A	528.4 ± 10.2	1.0	1.0	5.4	5.0
B	436.3 ± 9.6	0.5	6.7	4.3	4.0
C	524.8 ± 8.6	0.6	0.9	5.7	4.2
D	424.4 ± 7.8	0.7	0.8	4.0	3.3

CC_50_: mean concentration that killed 50% of cells; SI = ratio of cytotoxic to antiproliferative activity.
